# Research on the Optimization Method of Visual Sensor Calibration Combining Convex Lens Imaging with the Bionic Algorithm of Wolf Pack Predation

**DOI:** 10.3390/s24185926

**Published:** 2024-09-12

**Authors:** Qingdong Wu, Jijun Miao, Zhaohui Liu, Jiaxiu Chang

**Affiliations:** 1 School of Civil Engineering, Qingdao University of Technology, Qingdao 266520, China; sdlqwqd2024@126.com (Q.W.); msyu_990421@sina.com (J.M.); 2 Shandong Luqiao Group Co., Ltd., Jinan 250014, China; 3 School of Transportation, Shandong University of Science and Technology, Qingdao 266590, China; cjx745400@163.com

**Keywords:** camera calibration, optimization algorithm, CLI-WPP, reverse learning strategy, reprojection error

## Abstract

To improve the accuracy of camera calibration, a novel optimization method is proposed in this paper, which combines convex lens imaging with the bionic algorithm of Wolf Pack Predation (CLI-WPP). During the optimization process, the internal parameters and radial distortion parameters of the camera are regarded as the search targets of the bionic algorithm of Wolf Pack Predation, and the reprojection error of the calibration results is used as the fitness evaluation criterion of the bionic algorithm of Wolf Pack Predation. The goal of optimizing camera calibration parameters is achieved by iteratively searching for a solution that minimizes the fitness value. To overcome the drawback that the bionic algorithm of Wolf Pack Predation is prone to fall into local optimal, a reverse learning strategy based on convex lens imaging is introduced to transform the current optimal individual and generate a series of new individuals with potential better solutions that are different from the original individual, helping the algorithm out of the local optimum dilemma. The comparative experimental results show that the average reprojection errors of the simulated annealing algorithm, Zhang’s calibration method, the sparrow search algorithm, the particle swarm optimization algorithm, bionic algorithm of Wolf Pack Predation, and the algorithm proposed in this paper (CLI-WPP) are 0.42986500, 0.28847656, 0.23543161, 0.219342495, 0.10637477, and 0.06615037, respectively. The results indicate that calibration accuracy, stability, and robustness are significantly improved with the optimization method based on the CLI-WPP, in comparison to the existing commonly used optimization algorithms.

## 1. Introduction

Machine vision technology has widely penetrated many fields, such as industry, agriculture, medicine, and scientific research. In the image processing process of machine vision, it is often necessary to study the interrelationship between the 3D geometric position of a point on the surface of a space object and its corresponding point in the image [1] and then build the geometric model of camera imaging. The critical parameters of the camera can be obtained through experiments and complex calculations called camera calibration. In short, camera calibration aims to find a suitable mathematical model for converting from 3D to 2D, i.e., converting real-world images into a form amenable to mathematical computation and digital processing.

Currently, the main camera calibration techniques include the direct linear transformation method, the Tsai two-step calibration method, and Zhang Zhengyou’s calibration method [2,3,4,5], among which Zhang’s calibration method has been widely applied in practice. However, it relies on the nonlinear optimization algorithm, which has certain limitations and is prone to fall into local optima, resulting in the unsatisfactory accuracy of the calibration results. Therefore, domestic and foreign researchers have introduced intelligent optimization algorithms into camera calibration to eliminate the influence of initial value and enhance global search capability.

When exploring the application of traditional optimization algorithms in camera calibration techniques, a universal challenge lies in the instability of convergence performance, which often limits the efficiency and accuracy of the calibration process. To address the potential issue of poor convergence in traditional optimization algorithms during camera calibration, several studies [6,7] introduced genetic algorithms into the field of camera parameter calibration. Through complex operations such as gene encoding, decoding, crossover, and mutation, these algorithms aimed to enhance global search capabilities. However, while effective, the complexity of implementation and computational cost of this method cannot be ignored. To simplify the calibration process and accelerate it, the authors of [8,9] proposed a camera intrinsic parameter optimization strategy based on particle swarm optimization (PSO). This algorithm leverages the synergy between particles to approach the optimal solution during iterations. Nevertheless, a potential issue with PSO is the phenomenon of particle convergence, which can significantly reduce the diversity of the solution space, thereby affecting the accuracy and reliability of the calibration results. To tackle the limitation of calibration accuracy when the number of calibration images is limited, the authors of [10] ingeniously combined the advantages of simulated annealing and PSO algorithms. By simulating the temperature drop and equilibrium state search mechanisms in the physical annealing process, this approach aimed to improve the robustness and accuracy of the calibration process. Although this method improved calibration results to some extent, its gradual cooling and multiple iteration strategies also led to a slowdown in the overall convergence speed of the algorithm, increasing execution time. To further overcome the limitations in Zhang’s calibration method, the authors of [11] introduced the sparrow search algorithm (SSA) for camera calibration. The SSA enables information exchange and optimization iteration among individuals by simulating sparrow populations’ foraging and escape behaviors. However, the algorithm’s performance in adaptive adjustment mechanisms and global search capabilities remains to be enhanced, particularly for calibration tasks in complex scenarios.

To enhance the accuracy and efficiency of camera calibration, this paper innovatively introduces the Wolf Pack Predation (WPP) biomimetic algorithm as a calibration method. With its straightforward structure and minimal parameter adjustment requirements, the WPP algorithm significantly simplifies the implementation process and fundamentally reduces the risk of performance fluctuations caused by parameter misconfiguration. The core highlights of the WPP algorithm lie in its built-in adaptive adjustment mechanism and efficient information feedback system, which work in synergy to skillfully balance the algorithm’s local fine optimization and global extensive search capabilities, achieving a leap in calibration accuracy and a notable increase in convergence speed. Furthermore, the WPP algorithm deeply simulates the natural predation behavior of wolf packs, emphasizing the cooperative hunting and strategic guidance of alpha, beta, and delta wolves during the hunting process. This biomimetic design significantly strengthens the algorithm’s dual capabilities of global search and local optimization. When faced with complex camera calibration problems, the WPP algorithm demonstrates superior convergence performance compared to traditional methods, presenting remarkable performance in practical applications and revealing new possibilities in the field of camera calibration.

**Figure 1:**
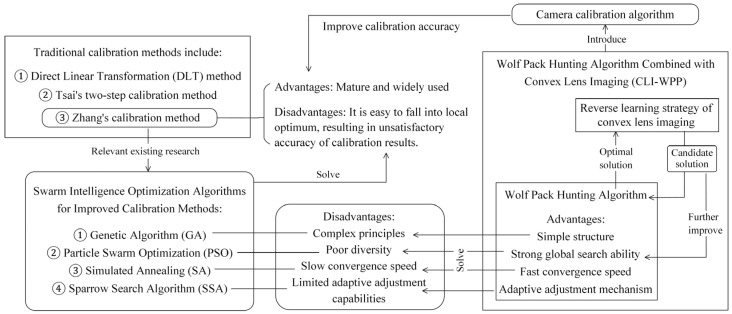
The logic diagram showing the novelty and advantages of the proposed method.

Additionally, considering the structural characteristics of optical lenses, this paper combines the advantages of the WPP algorithm with the imaging properties of convex lenses, aiming to obtain a more efficient and reliable new approach for optimizing camera calibration parameters. This paper innovatively introduces a reverse learning mechanism based on lens imaging. Specifically, as the WPP algorithm gradually approaches the optimal solution at a certain stage during iterations, we do not stop there but use this “optimal solution” as the starting point for lens imaging simulation. By simulating the refraction and focusing effects of convex lenses on light rays, we construct a virtual imaging process. In this process, the optimal solution is regarded as a light source or object point, and after the transformation effect of the lens, a series of new candidate solutions with expected better performance are formed on the image plane. These candidate solutions are not merely simple variations of the current optimal solution but, shaped by the principles of optical imaging, contain more global information and potential optimization directions. The introduction of these new solutions significantly enriches the search space, providing more exploration paths for the algorithm and enabling the optimization of vision sensor calibration parameters to more effectively escape from local optimal solutions and obtain more accurate and reliable optimization results. A logical diagram illustrating the novelty and advantages of the proposed method is shown in Figure 1.

In summary, the main contributions of this paper are as follows: We innovatively propose the CLI-WPP algorithm, which combines the Wolf Pack Predation biomimetic algorithm with the principles of convex lens imaging. By introducing a reverse learning strategy based on convex lens imaging to improve the algorithm of Wolf Pack Predation, the algorithm’s global optimal solution search capability is further enhanced. In addition, we introduce the CLI-WPP algorithm into the camera calibration algorithm, thus achieving the efficient optimization of camera calibration parameters and improving calibration accuracy. Lastly, the significant effects of the CLI-WPP camera calibration algorithm in enhancing calibration accuracy and efficiency are verified through comparative experiments. This research not only enriches the theoretical system of camera calibration techniques but also provides strong technical support for practical applications.

## 2. Nonlinear Imaging Model

**Figure 2:**
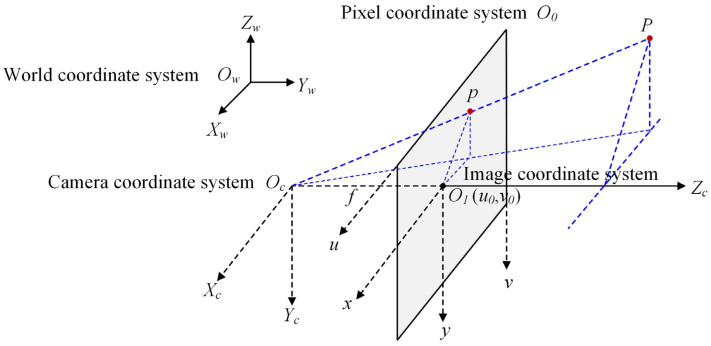
The relationship between the four coordinate systems.

The basic principle of camera calibration is to accurately convert the coordinates of an object or feature point in 3D space to the corresponding coordinates on a 2D image to obtain various camera parameters. This process involves four critical coordinate systems: the world coordinate system, the camera coordinate system, the image coordinate system, and the pixel coordinate system [12]. The relationship between the four coordinate systems during the mapping process of points in space is shown in Figure 2. In the figure, the world coordinate system is denoted by $O_{w}-X_{w}Y_{w}Z_{w}$, the camera coordinate system is denoted by $O_{C}-X_{C}Y_{C}Z_{C}$, the image coordinate system is denoted by $O_{1}-xy$, and the image pixel coordinate system is denoted by $O_{0}-uv$.

The first transformation converts world coordinates to camera coordinates. Let the coordinate of a point P in the scene be ($X_{w},\;{\;Y}_{w},\;\;Z_{w}$) in the world coordinate system and ($X_{c},\;\;Y_{c},\;\;Z_{c}$) in the camera coordinate system; the interrelated relationships can be expressed in the form of chi-square coordinates and matrices by rotating the matrix R and translating the vector T to achieve the corresponding transformation, as shown in Equation (1).

(1) $$\left[\begin{array}{c} X_{c}\\ Y_{c}\\ \begin{array}{c} Z_{c}\\ 1 \end{array} \end{array}\right]=\left[\begin{array}{cc} R & T\\ O^{T} & 1 \end{array}\right]\left[\begin{array}{c} x_{w}\\ y_{w}\\ \begin{array}{c} z_{w}\\ 1 \end{array} \end{array}\right]=M_{2}\left[\begin{array}{c} x_{w}\\ y_{w}\\ \begin{array}{c} z_{w}\\ 1 \end{array} \end{array}\right]\;,$$

In the above formula, *R* is a 3 × 3-unit orthogonal matrix; *T* is a 3 × 1 translation vector; $O={\left(0,\;\;0,\;\;0\right)}^{T}$; and the parameters *R* and *T* constitute the extrinsic parameters of camera calibration, $\left[\begin{array}{cc} R & T\\ O^{T} & 1 \end{array}\right]$, forming a 4 × 4 extrinsic matrix.

The second transformation converts camera coordinates to image coordinates [13]. The line between the imaging point P in the camera and the optical center $O_{c}$ of the camera is $O_{c}P$. The intersection of $O_{c}P$ and the image plane, denoted as p, is the projection of the spatial point P on the image plane. The coordinate of P in the coordinate system of the camera is ($X_{c},\;\;Y_{c},\;\;Z_{c}$), the coordinate of the point p in the coordinate system of the image is (x,  y), and the distance between the optical center and the projection point $O_{c}O_{1}$ is the focal length of the camera. According to Figure 1, the following Equation (2) can be obtained using the principle of similar triangles:

(2) $$\left\{\begin{array}{c} \frac{x}{X_{c}}=\frac{f}{Z_{c}}\\ \frac{y}{Y_{c}}=\frac{f}{Z_{c}} \end{array}\right.,$$

The camera coordinates are converted to image coordinates in the form of chi-square coordinates and matrices, as shown in Equation (3):

(3) $$Z_{c}\left[\begin{array}{c} x\\ y\\ 1 \end{array}\right]=\left[\begin{array}{cc} f & 0\\ 0 & f\\ 0 & 0 \end{array}\right.\left.\begin{array}{cc} 0 & 0\\ 0 & 0\\ 1 & 0 \end{array}\right]\left[\begin{array}{c} X_{c}\\ Y_{c}\\ \begin{array}{c} Z_{c}\\ 1 \end{array} \end{array}\right],$$

The third transformation is the conversion of image coordinates to pixel coordinates. The pixel coordinate system [14] ${uO}_{0}v$ is a two-dimensional right-angled coordinate system with the origin $O_{0}$ located in the upper left corner of the image, and the u-axis and v-axis intersect at the origin. The origin of the image coordinate system is located at the intersection of the camera’s optical axis and the image plane (called the principal point), i.e., the center of the image $O_{1}$. The x-axis and y-axis are parallel to the u-axis and v-axis, respectively. If the coordinate of $O_{1}$ in the pixel coordinate system is $\left(u_{0},\;\;v_{0}\right)$, and the physical dimensions of each pixel in the image in the x and y directions are dx and dy, respectively, then the relationship between the pixel coordinate (u,  v) and the image coordinate (x,  y) at the point on the image is shown in Equation (4) as follows:

(4) $$\left\{\begin{array}{c} u=\frac{x}{dx}+u_{0}\\ v=\frac{y}{dy}+v_{0} \end{array}\right.,$$

The image coordinates are converted to pixel coordinates in the form of homogeneous coordinates and matrices, as shown in Equation (5).

(5) $$\left[\begin{array}{c} u\\ v\\ 1 \end{array}\right]=\left[\begin{array}{ccc} 1/dx & 0 & u_{0}\\ 0 & 1/dy & v_{0}\\ 0 & 0 & 1 \end{array}\right]\left[\begin{array}{c} x\\ y\\ 1 \end{array}\right],$$

In this equation, dx and dy represent the physical sizes of pixels in the x-axis and y-axis directions, respectively, while $\left(u_{0},\;\;v_{0}\right)$ is the coordinate of the principal point, which is also known as the image origin.

By substituting Equations (3) and (5) into Equation (1), the relationship between point p in the image pixel coordinate system and the world coordinate point P can be calculated.

(6) $$Z_{c}\left[\begin{array}{c} u\\ v\\ 1 \end{array}\right]=\left[\begin{array}{ccc} \frac{1}{dx} & 0 & u_{0}\\ 0 & \frac{1}{dy} & v_{0}\\ 0 & 0 & 1 \end{array}\right]\left[\begin{array}{cc} f & 0\\ 0 & f\\ 0 & 0 \end{array}\right.\left.\begin{array}{cc} 0 & 0\\ 0 & 0\\ 1 & 0 \end{array}\right]\left[\begin{array}{cc} R & t\\ 0 & 1 \end{array}\right]\left[\begin{array}{c} x_{w}\\ y_{w}\\ \begin{array}{c} z_{w}\\ 1 \end{array} \end{array}\right]=\left[\begin{array}{cc} a_{x} & 0\\ 0 & a_{y}\\ 0 & 0 \end{array}\right.\left.\begin{array}{cc} u_{0} & 0\\ v_{0} & 0\\ 1 & 0 \end{array}\right]\left[\begin{array}{cc} R & T\\ 0^{T} & 1 \end{array}\right]\left[\begin{array}{c} x_{w}\\ y_{w}\\ \begin{array}{c} z_{w}\\ 1 \end{array} \end{array}\right]=M_{1}M_{2}X_{W}=MX_{W}$$

where $a_{x}=f/dx$ and $a_{y}=f/dy$ are called the scale factors of the u and v axes; they denote the pixel units of the camera in the x-direction and y-direction, respectively. $M_{1}$ is the internal parameter matrix of the camera, $M_{2}$ is the external parameter matrix of the camera, and M is the projection matrix.

**Figure 3:**
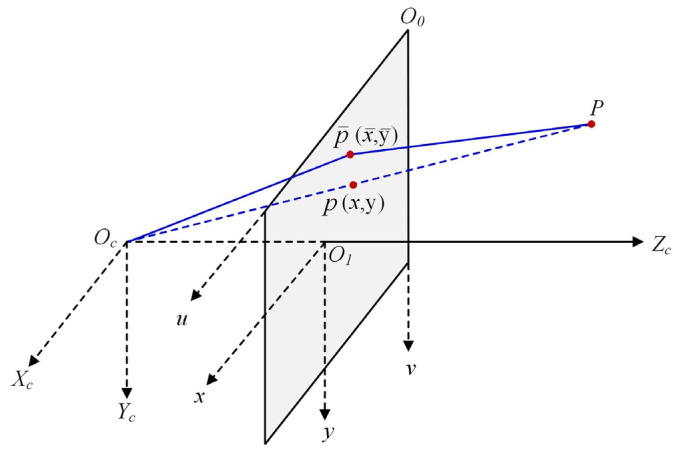
Position shift phenomenon due to distortion.

The above formula is the imaging result without any interference. In practice, there is a certain degree of deviation between the actual imaging position of an object or a feature point and the ideal imaging position, and the camera’s nonlinear optical aberration causes this deviation. Figure 3 shows the positional shift due to aberrations.

As can be seen from Figure 3, point P on the world coordinate system has an ideal imaging point p (x, y) on the image coordinate system. However, because of camera distortion, the actual imaging position is shifted to $\bar{p}(\bar{x}$, $\bar{y})$. To shift the point due to camera aberration as close as possible to the mapped point in the ideal state, the aberration coefficient generated by the camera must be corrected. Zhang Zhengyou’s calibration method only considers radial aberrations, which greatly influence the model, and it does not consider tangential aberrations. The radial distortion coefficient can be derived from the numerical relationship between the real and ideal coordinates; see Equation (7).

Real image coordinates $(\bar{x}$, $\bar{y})$ in relation to ideal image coordinates (x, y) are determined using the following equation (Taylor expansion):

(7) $$\left\{\begin{array}{c} \bar{x}=x+x[k_{1}(x^{2}+y^{2})+k_{2}{\left(x^{2}+y^{2}\right)}^{2}]\\ \bar{y}=y+y{[k}_{1}(x^{2}+y^{2})+{k_{2}(x^{2}+y^{2})}^{2}] \end{array}\right.,$$

By substituting Equation (4) into Equation (7), we obtain the relationship between the actual pixel coordinates $(\bar{u}$,$\;\bar{v})$ and the ideal pixel coordinates (u,v) as follows:

(8) $$\left\{\begin{array}{c} \bar{u}=u+\left(u-u_{0}\right)\left[k_{1}\left(x^{2}+y^{2}\right)+k_{2}{\left(x^{2}+y^{2}\right)}^{2}\right]\\ \bar{v}=v+\left(v-v_{0}\right)\left[k_{1}\left(x^{2}+y^{2}\right)+k_{2}{\left(x^{2}+y^{2}\right)}^{2}\right] \end{array}\right.,$$

(9) $$\left[\begin{array}{c} \left(u-u_{0})(x^{2}+y^{2}\right)\\ \left(v-v_{0})(x^{2}+y^{2}\right) \end{array}\right.\left.\;\;\begin{array}{c} (u-u_{0}){\left(x^{2}+y^{2}\right)}^{2}\\ \left(v-v_{0})(x^{2}+y^{2}\right) \end{array}\right]\left[\begin{array}{c} k_{1}\\ k_{2} \end{array}\right]=\left[\begin{array}{c} \bar{u}-u\\ \bar{v}-v \end{array}\right],$$

where $\left(\bar{x},\;\bar{y}\right)$ denotes the actual image coordinates after distortion, and $\left(\bar{u},\;\bar{v}\right)$ denotes the actual pixel coordinate after distortion; (*x*, *y*) is the image coordinate in the ideal case without distortion, and (u,  v) refers to the corresponding pixel coordinate in the ideal case without distortion; $k_{1}$ and $k_{2}$ represent the radial distortion coefficients.

**Figure 4:**
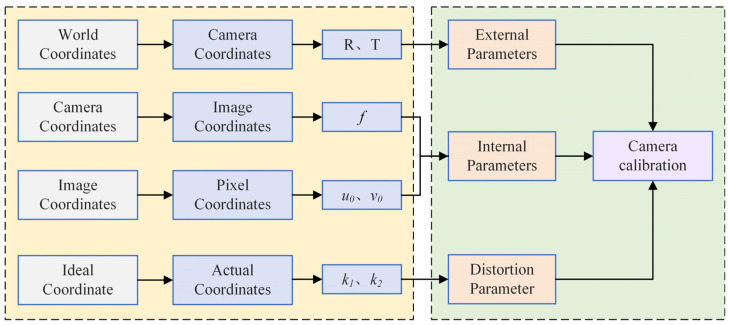
Relationship of coordinate transformation in camera calibration.

In summary, the coordinate change relationship in camera calibration can be summarized as follows: The world coordinates are converted to camera coordinates to determine the external parameters of camera calibration, i.e., translation matrix T and rotation matrix R. Then, the camera coordinates are converted to image coordinates to obtain the focal length f of the camera, and the image coordinates are converted to pixel coordinates to determine the coordinates of the camera’s principal point $\left(u_{0},v_{0}\right)$. Lastly, the ideal coordinates are converted to actual coordinates to determine the camera’s distortion coefficients $k_{1}$ and $k_{2}$; thus, camera calibration is completed. The coordinate change relationship is shown in Figure 4.

## 3. Principle of the Bionic Algorithm of Wolf Pack Predation (WPP)

The bionic algorithm of Wolf Pack Predation (WPP) is a pack intelligence optimization algorithm inspired by the social hierarchical stratification and predation behavior in wolf packs [15]. In wolf society, there is a strict hierarchy in which the α wolf is at the highest rank and is responsible for overall decision-making; the β wolf follows closely behind, assisting the α wolf and directing other members; the δ wolf is at the third rank, carrying out orders and accountable for scouting; and the ω wolf is at the lowest rank and is responsible for balancing the intra-population relationships. In the WPP algorithm, each wolf is regarded as a candidate solution, representing possible solutions to the optimization problem. Among them, the α wolf corresponds to the optimal solution, the β wolf corresponds to the second-best solution, and the δ wolf corresponds to the third-best solution. In contrast, the other wolves are regarded as ω solutions. These groups of wolves collaborate to search for and track their prey, usually with the α wolf leading the β wolf and the δ wolf in a siege. If the prey escapes, the other wolves continue the pursuit until the prey is captured. The WPP algorithm simulates this behavioral pattern of wolf packs and uses the collaborative strategy of wolf packs to solve various optimization problems.

The mathematical model of a wolf pack encircling its prey is as follows:

(10) $$D=\left|C\cdot X_{p}(t)-X(t)\right|,$$

(11) $$X(t+1)=X_{p}(t)-A\cdot D,$$

Equation (10) can determine the relative displacement D between the individual and the prey, and Equation (11) can be used to achieve the localization tracking of the wolf. In the above equation, X and $X_{p}\;$are the position vectors of the wolf individual and prey, respectively, t is the current iteration number, and A and C are called coefficient vectors, which are determined as follows:

(12) $$A=2a\cdot r_{1}-a,$$

(13) $$C=2\times r_{3}-a,$$

where the magnitude of $r_{1}$ is taken as a random number in the range of [0, 1] [16]; the magnitude of $r_{3}$ is taken as a random number in the range of [0.5, 1.5]; a is a convergence factor, and the value of this convergence factor decreases linearly with the number of iterations; decreases linearly from 2; and eventually, the value of this convergence factor will reach 0.

The mathematical model of a wolf attacking its prey is as follows:

(14) $$X_{1}=X_{\alpha }-A_{1}\cdot \left|C_{1}\cdot X_{\alpha }-X\right|,$$

(15) $$X_{2}=X_{\beta }-A_{2}\cdot \left|C_{2}\cdot X_{\beta }-X\right|,$$

(16) $$X_{3}=X_{\delta }-A_{3}\cdot \left|C_{3}\cdot X_{\delta }-X\right|,$$

(17) $$X(t+1)=\frac{X_{1}(t)+X_{2}(t)+X_{3}(t)}{3},$$

where $X_{\alpha }$, $X_{\beta }$, and $X_{\delta }$ are the position vectors of α, β, and δ wolves, respectively; $A_{1}$, $A_{2}$, and $A_{3}$ are the position vectors of A; and $C_{1}$, $C_{2}$ and $C_{3}$ the position vectors of C.

## 4. CLI-WPP

Addressing the limitation of the bionic algorithm of Wolf Pack Predation in escaping from local optima, we adopted an improved approach. Firstly, a reverse learning strategy based on convex lens imaging technology is introduced to enhance performance. Then, utilizing this strategy, we transform the current optimal individual obtained by the bionic algorithm of Wolf Pack Predation, generating a series of new individuals that are different from the original ones but possess potentially better solutions. These new individuals not only expand the search space but also assist the algorithm in escaping from the confinement of local optima. Ultimately, this strategy enables the algorithm to explore global optimal solutions further.

### 4.1. The Law of Imaging by a Convex Lens

**Figure 5:**
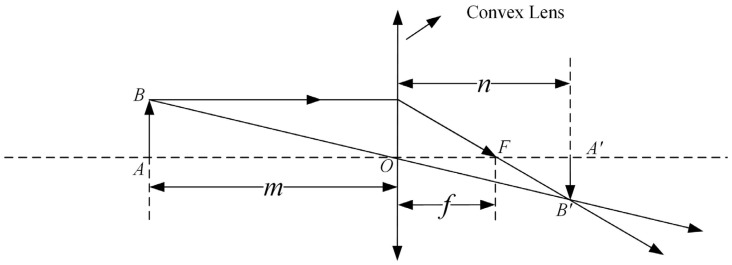
Schematic diagram of convex lens imaging of light.

The law of imaging by a convex lens [17] is a fundamental principle in optics, which states that when an object is placed beyond its focal length, an inverted image will be formed on the other side. This principle is illustrated in Figure 5.

The formula for the imaging of a convex lens can be derived from Figure 5 as follows:

(18) $$\frac{1}{m}+\frac{1}{n}=\frac{1}{f},$$

where m is the object distance, n is the image distance, and f is the focal length of the lens.

### 4.2. Reverse Learning Strategies for Convex Lens Imaging Techniques

**Figure 6:**
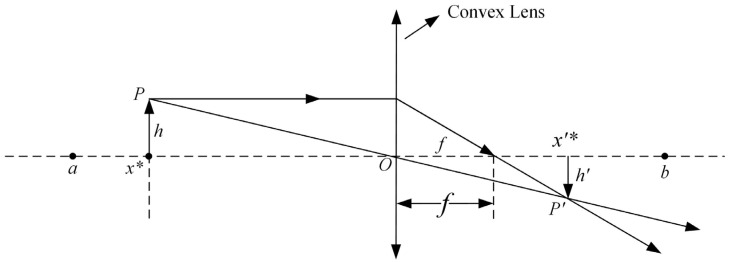
The reverse learning strategy for the convex lens imaging technique.

As shown in Figure 6, taking one-dimensional space as an example, the search range of the solution on the *x*-axis is [a, b], and the center of the search range is the *y*-axis where the convex lens is located. Suppose there is a body P projected on the *x*-axis as $x^{*}$ with height h. A real image P′ can be obtained by imaging through the convex lens, and P′ is projected on the *x*-axis as ${x\prime }^{*}$, with height *h’*. From this, the inverse of the individual $x^{*}$ can be obtained as the individual ${x\prime }^{*}$.

In Figure 6, the globally optimal individual $x^{*}$ is used to determine its corresponding inverse point ${x\prime }^{*}$ with O as the base point, which can be derived from the convex lens imaging principle as follows:

(19) $$\frac{\frac{a+b}{2}-x^{*}}{{x\prime }^{*}-\frac{a+b}{2}}=\frac{h}{h\prime },$$

Let h/h′=k. Equation (19) is transformed to obtain the solution formula ${x\prime }^{*}$ to determine the inverse learning solution using the convex lens imaging method as follows:

(20) $${x\prime }^{*}=\frac{a+b}{2}+\frac{a+b}{2k}-\frac{x^{*}}{k},$$

### 4.3. Camera Calibration Using CLI-WPP

The bionic algorithm of WPP demonstrates the high speed and high quality of optimality finding when dealing with low-dimensional functions. The α, β, and δ wolf are at a local optimum. A large number of wolves will gather at a particular location. This makes it difficult for the bionic algorithm of WPP to move away from the local optimum. For four-dimensional or more complex search tasks, such as camera calibration, the search efficiency of the WPP algorithm is severely weakened. At the same time, this can also drastically reduce its accuracy.

**Figure 7:**
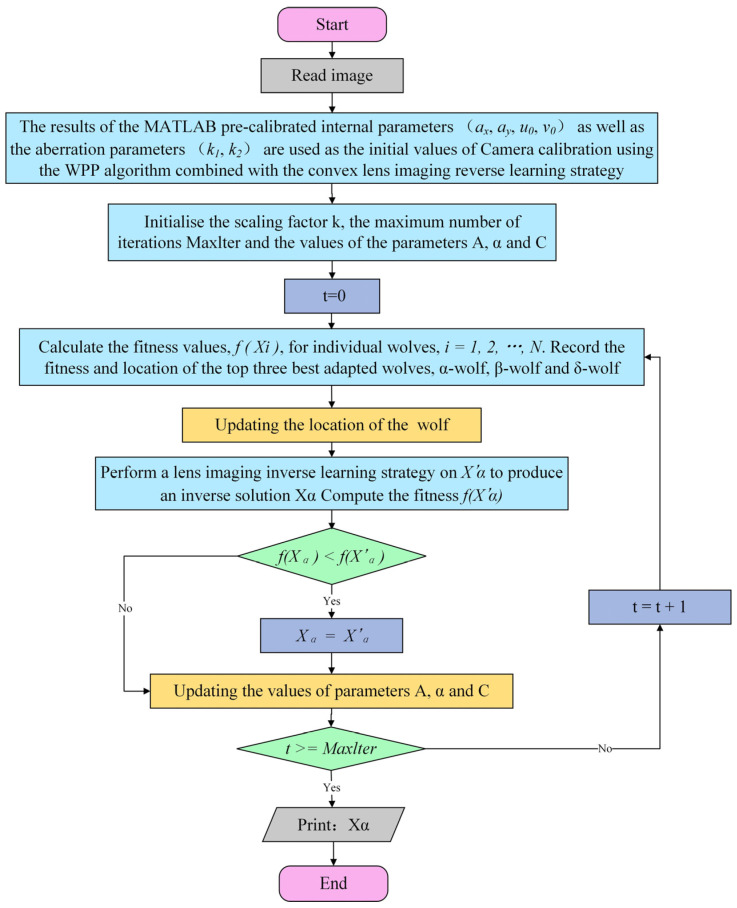
Flowchart of the optimized camera calibration method using the CLI-WPP.

By leveraging convex lens imaging techniques and adopting a reverse learning strategy, we create a series of new individuals that differ from the original but may outperform it. These new individuals not only broaden the search horizon but also empower the algorithm to escape the confines of local optima and delve deeper into the prospects of a globally optimal solution. As a result, we integrate the inverse learning strategy of convex lens imaging with the bionic algorithm of Wolf Pack Predation (CLI-WPP) to streamline and enhance the calibration process. The implementation steps of this improved WPP algorithm are outlined in Figure 7.

The specific steps are as follows:

Step 1. Read the image.

Step 2. Based on Zhang Zhengyou’s calibration technique, using MATLAB R2016b software, calculate the initial parameters of the camera to be tested, including the internal parameters ($a_{x}$,${\;a}_{y}$,$\;u_{0}$,${\;v}_{0}$) and distortion parameters ($k_{1}$, $k_{2}$), as well as the external parameters (R,T). The pre-calibrated internal parameters and distortion parameter results are used as the initial values for the convex lens imaging reverse learning strategy and the bionic algorithm of Wolf Pack Predation. 

Step 3. Initialize the scaling factor k; the iteration count t; the maximum iteration count Maxlter; and the values of parameters A, α, and C. 

Step 4. Construct the objective function using the average reprojection error as follows:

(21) $$f\left(X_{i}\right)=F\left(a_{x},a_{y},u_{0},\;v_{0},k_{1},k_{2}\right)=\frac{1}{m}\sum_{i=1}^{m}\sqrt{{\left(\bar{u_{i}}-u_{i}\right)}^{2}+{\left(\bar{v_{i}}-v_{i}\right)}^{2}},$$

In the above formula, m is the number of checkerboard corner points, ($\bar{u},\bar{v}$) is the image pixel coordinate extracted by the corner detection algorithm, and (u,v) is the image pixel coordinates obtained after projecting the 3D points using the camera model. The goal of the optimization is to make the difference (i.e., the reprojection error) between the image pixel coordinates ($\bar{u},\bar{v}$) extracted by the corner detection algorithm and the image pixel coordinates ($\bar{u},\bar{v}$) obtained by projecting the 3D points using the camera model as small as possible.

Step 5. Record the current iteration count; calculate the fitness value $f(X_{i})$ of each individual; record the fitness values and individual positions of the α wolf, the β wolf, and the δ wolf for this iteration; and update the positions of the current wolves.

Step 6. Apply the convex lens imaging reverse learning strategy to Xα based on Equation (20) to generate the reverse solution $X^{\prime }\alpha$, as shown in Equations (22) and (23).

(22) $$X^{\prime }\alpha =\left({x\prime }_{1},{x\prime }_{2},\cdots ,{x\prime }_{j}\right),\;\;j=1,2,\cdots D,$$

(23) $${x\prime }_{j}=\frac{a_{j}+b_{j}}{2}+\frac{a_{j}+b_{j}}{2k}-\frac{x_{j}}{k},$$

In these equations, ${x\prime }_{j}$ and $x_{j}$ represent the jth dimensional components of x′ and x, respectively, while $a_{j}$ and $b_{j}$ represent the jth dimensional components of the upper and lower bounds of the decision variables. If $f\left(X_{\alpha }\right)<f\left({X^{\prime }}_{\alpha }\right)$, then the reverse solution ${X\prime }_{\alpha }$ replaces $X_{\alpha }$; otherwise, proceed directly to Step 7. 

Step 7. Update the values of parameters A, α, and C.

Step 8. Check if the maximum number of iterations is reached. If satisfied, output the optimal solution Xα; the result corresponding to Xα is the required camera calibration parameter. Otherwise, increase the iteration count and return to Step 5.

## 5. Experiment and Analysis

**Figure 8:**
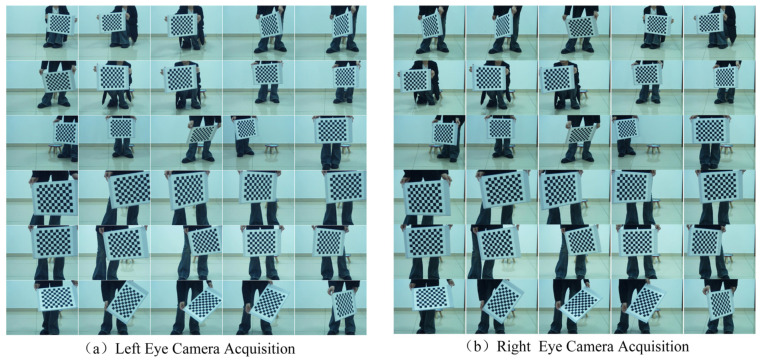
Calibration plate image acquisition.

**Figure 9:**
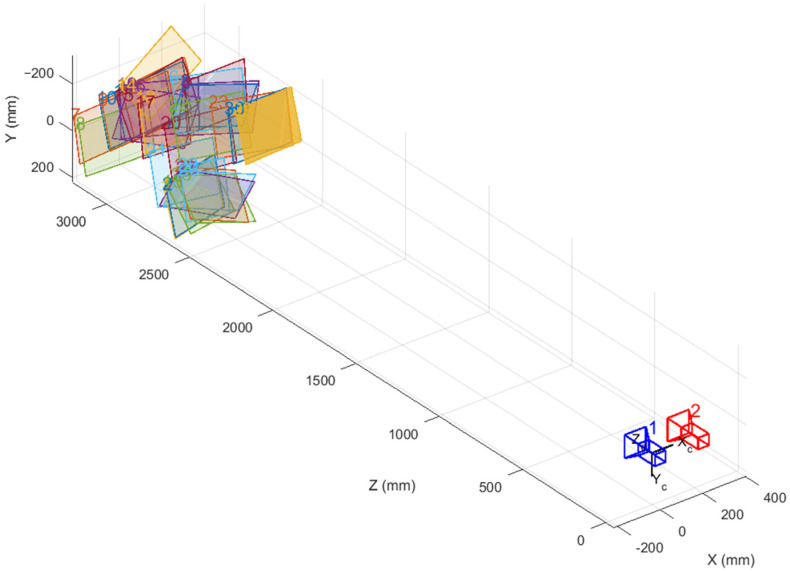
The 3D model diagram of the external parameters of the camera.

To validate the performance of the improved algorithm, a camera calibration experiment was conducted using the MATLAB calibration toolbox. The experiment utilized a binocular camera (Brand: Pixel XYZ, model: D-455B, Pixel Leap (Wuhan) Technology Co., Ltd., Wuhan, China) to capture images. During the experiment, the camera was securely mounted on a stable stand to minimize the impact of vibrations on image quality. The chessboard calibration plate image employed a standard 9 × 12 black-and-white grid design, with each grid measuring 30 × 30 mm, to ensure adequate feature points for algorithm recognition. During the photography process, we captured thirty photos featuring the chessboard grid in the same scene, covering various angles and camera positions to test the robustness of our method thoroughly. Although all pictures were taken indoors, the indoor calibration environment did not prevent the application of the calibrated camera from outdoor real-world scenes, as illustrated in Figure 8. Pre-calibration was performed using the MATLAB calibration toolbox, and the calibration results for internal and distortion parameters are listed in Table 1. The calibration results for external parameters are presented in Table 2. The spatial positional relationship between the left and right cameras and each checkerboard image is illustrated in Figure 9.

**Figure 10:**
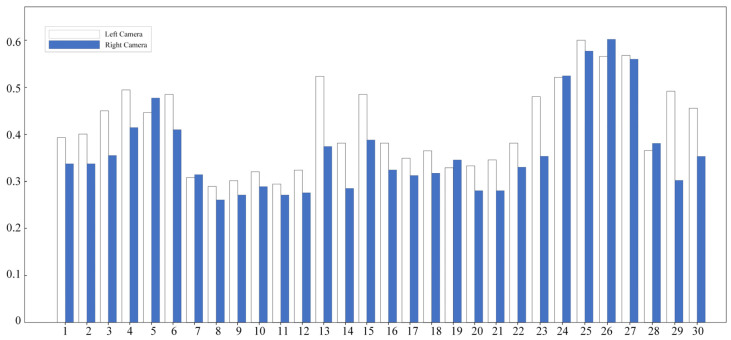
Binocular camera reprojection error graph. The horizontal coordinates of the figure indicate the 30 images used for left and right target timing, and the vertical coordinates indicate the value of the reprojection error.

The internal parameters and distortion parameters for the left and right cameras are $a_{x},a_{y},u_{0},v_{0},k_{1},{and\;k}_{2}$, and these results were used as the initial values for the CLI-WPP. The histogram of reprojection errors of the left and right cameras after preliminary calibration is shown in Figure 10. The preliminary calibration was accomplished using Zhang’s calibration method, and its reprojection error was relatively high, at 0.42986500. The relatively high reprojection error of 0.42986500 was primarily attributed to the use of all images captured during the experiment, which inevitably included some images with poor quality due to various factors such as occlusions, and fuzzy and indistinct images. These images introduced additional errors into the calibration process, thereby affecting the reprojection error result. To obtain more accurate camera parameters and reduce the reprojection error while ensuring that the number of images meets the requirements, we implemented a rigorous screening of the images. The screening was based on Figure 10, where images with large reprojection errors indicated in the histogram were eliminated, aiming to achieve more precise camera parameters and reprojection errors using Zhang’s calibration method. As listed in Table 5, after screening and recalibration, we achieved a lower reprojection error value of 0.28847656, indicating that the camera parameters were estimated with greater accuracy.

Through several experiments, the smallest average reprojection error values were obtained when the optimized calibrated left and right eyepiece camera parameters were based on the CLI-WPP algorithm, as shown in the data listed in Table 3 and Table 4, respectively.

## 6. Result Analysis and Discussion

To evaluate the accuracy of the proposed algorithm in this paper, the calibration optimization method (CLI-WPP) was compared and analyzed with camera calibration methods based on simulated annealing (SA), Zhang’s calibration method, the sparrow search algorithm (SSA), particle swarm optimization (PSO), and the bionic algorithm of Wolf Pack Predation (WPP). A controlled variable method was adopted to avoid the uncertainty caused by optimizing too many parameters simultaneously. Here, the camera’s external parameters were fixed, while the internal and distortion parameters were optimized. After obtaining the initial values of the internal parameters and distortion parameters, they were extended in the following manner to create search boundaries for each parameter [18]: [$a_{x},a_{y},u_{0},v_{0}$] ± 100, [$k_{1},\;k_{2}$]∈[−1, 1]. In the experiment, the population size was set to 50, and the maximum number of iterations was 200.

After optimization with the algorithms above, the calibrated parameters for the left camera were determined, which are listed in Table 5. Based on these parameters, the minimum average reprojection error value $f_{err}$ was obtained.

**Table 5:** Optimization results of various algorithms for the left camera’s parameters.

Parameters	SA	Zhang’s	SSA
$a_{x}$	3322.09000	3305.65809	3363.40540
$a_{y}$	3325.41000	3323.02436	3400.48806
$u_{0}$	610.020000	601.474397	578.705639
$v_{0}$	443.970000	434.541164	432.936919
$k_{1}$	0.11770000	−0.09525949	−0.99725691
$k_{2}$	1.12440000	0.32817828	−0.29592964
$f_{err}$	0.42986500	0.28847656	0.23543161
**Parameters**	**PSO**	**WPP**	**CLI-WPP**
$a_{x}$	3303.18672	3300.95863	3297.40374
$a_{y}$	3308.39923	3318.14506	3321.59384
$u_{0}$	576.638947	580.395389	5.79399989
$v_{0}$	432.069792	429.931569	4.30634941
$k_{1}$	0.41377230	−0.05267407	−0.01744174
$k_{2}$	−1.00000000	0.5.6781038	0.38125975
$f_{err}$	0.21934249	0.10637477	0.06615037

As seen from the data in Table 5, the mean reprojection error of SA is 0.42986500, the same as the pre-calibration value in MATLAB. The mean reprojection errors of Zhang’s calibration method, the SSA, and PSO are 0.28847656, 0.23543161, and 0.219342495, respectively, all showing a decrease compared to the pre-calibration values. The mean reprojection error of the WPP algorithm is reduced to 0.10637477 after optimization, demonstrating the effectiveness of the WPP algorithm in improving calibration accuracy. When using the CLI-WPP algorithm, the mean reprojection error is significantly reduced to 0.06615037, indicating the superior performance of the CLI-WPP algorithm in camera calibration.

**Figure 11:**
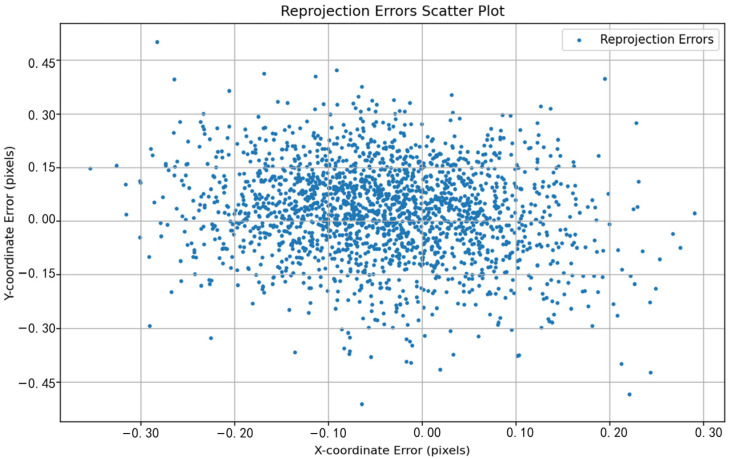
The reprojection error distribution of SA.

**Figure 12:**
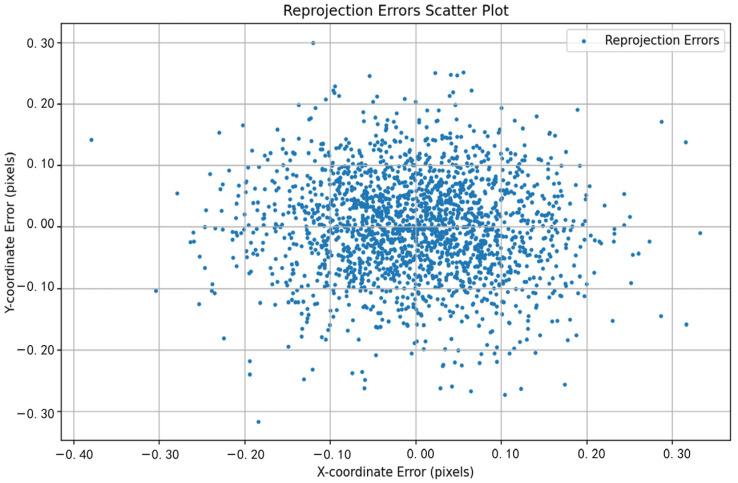
The reprojection error distribution of Zhang’s calibration method.

**Figure 13:**
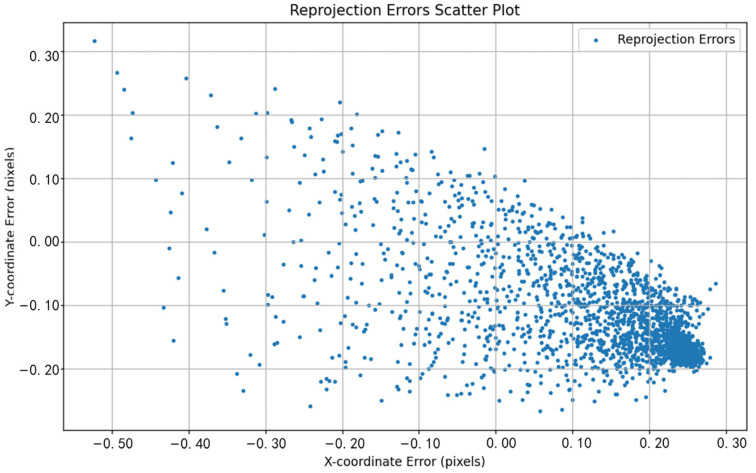
The reprojection error distribution of PSO.

**Figure 14:**
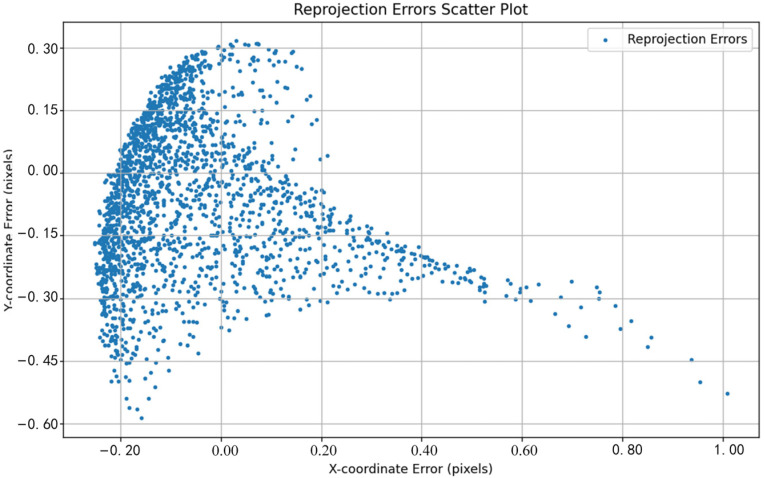
The reprojection error distribution of the SSA.

**Figure 15:**
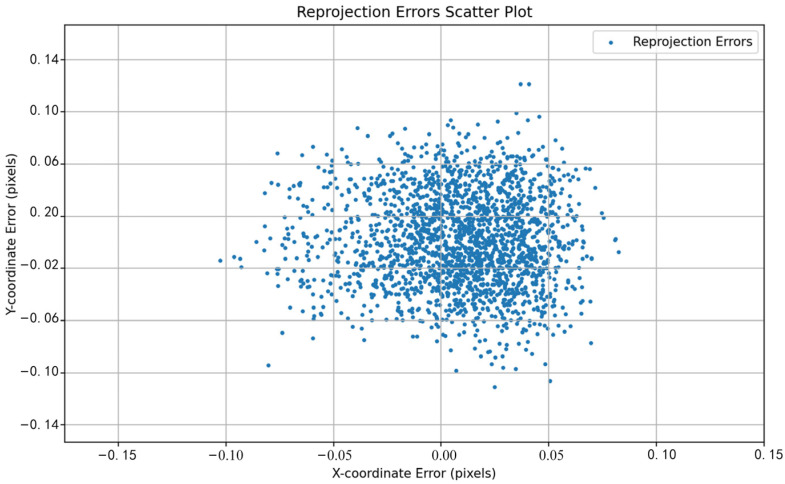
The reprojection error distribution of WPP.

**Figure 16:**
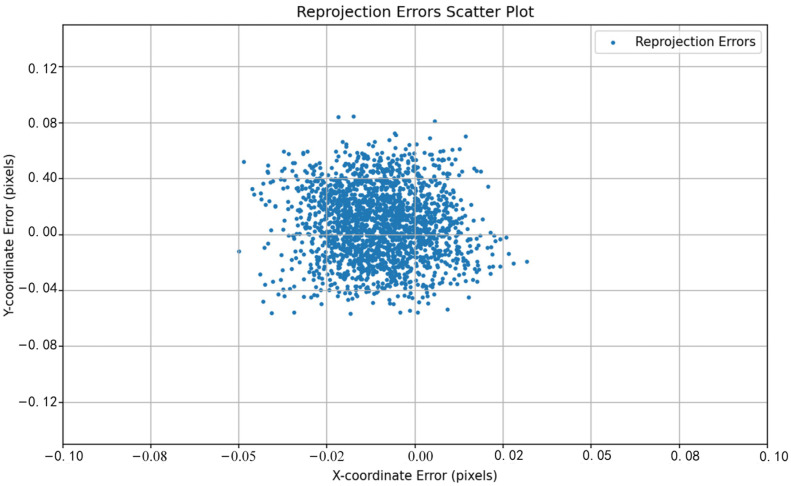
The reprojection error distribution of the CLI-WPP.

The performance differences between different methods can be visually observed by comparing the reprojection error distributions shown in Figure 11, Figure 12, Figure 13, Figure 14, Figure 15 and Figure 16. Figure 11 represents the calibration results of the SA, which has a mean reprojection error of 0.42986500, the same as the pre-calibration value in MATLAB. By comparing Figure 12 with Figure 11, it can be seen that after removing images with larger reprojection errors, the reprojection error obtained by Zhang’s calibration method is significantly reduced. Comparing Figure 12 with Figure 13 and Figure 14, it can be observed that PSO and the SSA tend to get stuck in local optimal solutions during the optimization process, and the deviations between local optimal solutions are relatively large. When comparing Figure 15 with Figure 11 and Figure 12, it can be seen that the reprojection error distribution after WPP optimization is more concentrated than that of SA and Zhang’s calibration method, indicating a further improvement in calibration accuracy. By comparing Figure 16 with Figure 15, it can be seen that the CLI-WPP algorithm significantly improves optimization performance compared to the WPP algorithm. As shown in Figure 16, the reprojection error distribution density after CLI-WPP optimization is the smallest, demonstrating the high accuracy of its calibrated parameters.

**Figure 17:**
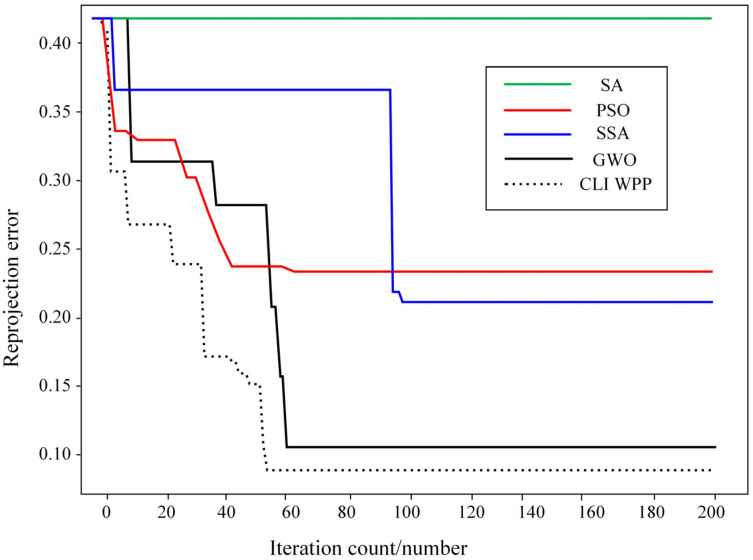
The objective value optimization process.

Figure 17 depicts the changes in the objective value during the optimization process of camera calibration for the SA, SSA, PSO, WPP, and CLI-WPP. It can be observed that the camera calibration method based on SA (represented by the green line) has poor iterative ability and remains in its initial state. The camera calibration method based on the SSA (blue line) has a slow iterative speed and falls into a local optimum after the 100th iteration, with no further optimization. The camera calibration method based on PSO (red line) performs well initially but stabilizes after 65 iterations. The calibration method based on the WPP algorithm (black line) stabilizes after approximately 60 iterations. However, the camera calibration method based on the CLI-WPP (dashed line) stabilizes after only 50 iterations, and the CLI-WPP method achieves the most minor reprojection error. The CLI-WPP algorithm converges faster, with a more significant step-down in the optimization value, indicating that it is more effective in escaping local optima during the search process and finding the global optimum faster.

In contrast, the WPP algorithm requires more iterations to reach a stable state, which may suggest that it encounters local optima during the search process. It also requires more iterations to escape these local optimal regions. Through comparative experiments, it is evident that the CLI-WPP algorithm can effectively handle local optima issues, escape local optima, find the global optimum, and achieve significantly improved convergence efficiency while being more robust and reliable. Furthermore, as shown in Figure 17, the average reprojection error obtained with the CLI-WPP algorithm is 0.034 smaller than that obtained with the WPP algorithm, indicating that the CLI-WPP calibration method has superior accuracy.

**Figure 18:**
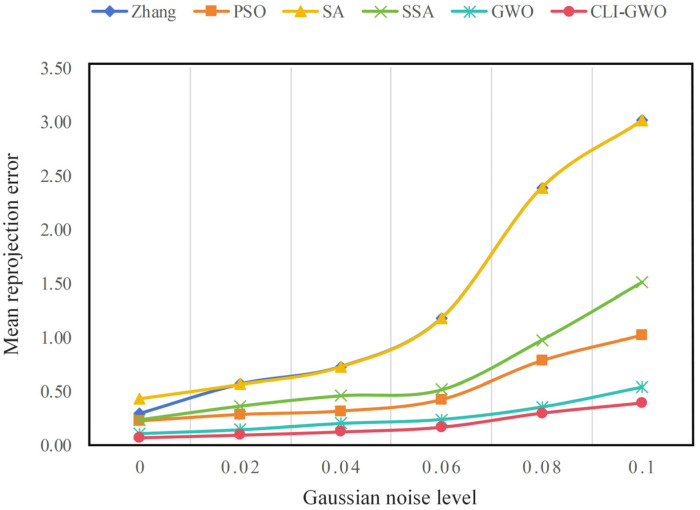
Average calibration results under different levels of noise intensity.

To evaluate the robustness of the proposed algorithm in this paper, Gaussian noise with a mean of 0 and variances of 0.02, 0.04, 0.06, 0.08, and 0.10 was added to the 30 captured images of a checkerboard calibration board. The simulated annealing (SA) algorithm, Zhang’s calibration method, sparrow search algorithm (SSA), particle swarm optimization (PSO), the bionic algorithm of Wolf Pack Predation (WPP), and the proposed method in this paper were used to conduct camera calibration experiments in turn. The calibration results were averaged over 50 independent experiments for each specified variance value. As shown in Figure 18, a coordinate system was established with different Gaussian noise values on the *x*-axis and the average reprojection error on the *y*-axis. It can be intuitively observed that as the noise increases, the calibration accuracy of all six methods decreases. Still, the decline using the proposed method is less than that of the other methods. This indicates that the camera calibration method based on the CLI-WPP exhibits excellent robustness within a specific range of noise variations.

## 7. Conclusions

Addressing the issues of insufficient accuracy and poor robustness in current standard camera calibration methods, we proposed the CLI-WPP for camera calibration. Firstly, the basic principles of camera calibration, the working principles of WPP, as well as CLI-WPP, were elaborated. By applying the CLI-WPP algorithm to optimize the internal parameters of the camera, high-precision camera calibration was achieved.

To verify the effectiveness of the CLI-WPP algorithm, we conducted a series of experimental comparisons with the SA, SSA, PSO, and WPP methods. The experimental results show that the CLI-WPP-based camera calibration method significantly improves accuracy and robustness compared to other optimization algorithms. By enhancing the accuracy and robustness of camera calibration, the proposed method in this paper provides more effective means and approaches for camera calibration research and practical applications.

While this study has achieved remarkable results in demonstrating the effectiveness of the CLI-WPP algorithm through the critical indicator of reprojection error, we also acknowledge the limitations and room for improvement of this method. Firstly, the current evaluation system primarily relies on the single metric of reprojection error, which may not fully capture the precision and performance of the calibration experiments. To address this limitation, we plan to incorporate more diversified evaluation metrics in our future research, aiming to establish a more comprehensive and robust framework for assessing the precision of calibration experiments.

Furthermore, we recognize that the complexity of application scenes often influences the precision and performance of calibration experiments. Therefore, exploring evaluation methods for calibration experiment precision in different application scenes will be an essential direction for future research. This will help us gain a deeper understanding of the performance of calibration algorithms in practical applications and provide strong support for their optimization.

In summary, we anticipate that future research will introduce more diversified evaluation metrics; explore assessment methods for various application scenes; and continuously optimize the CLI-WPP algorithm to establish a more comprehensive, accurate, and adaptable calibration experiment precision evaluation system. This will further develop camera calibration technology and provide more solid technical support for practical applications in related fields.

## Figures and Tables

**Table 1:** Calibration results for internal parameters and distortion parameters of the left and right cameras.

Parametric	Left Camera	Right Camera
$a_{x}(pixel)$	3322.09000	3314.01000
$a_{y}(pixel)$	3325.41000	3314.72000
$u_{0}(pixel)$	610.020000	619.060000
$v_{0}(pixel)$	443.970000	405.520000
$k_{1}$	0.11770000	0.04220000
$k_{2}$	1.12440000	0.41570000

**Table 2:** Calibration results of the external parameters of the left and right cameras.

Parametric	Result		
Translation Matrix	−203.505423	−1.12304825	8.51035436
Rotation Matrix	0.99975432	0.00493237	0.01674298
	0.00513486	0.99989998	0.01275354
	0.01664542	0.01294541	0.99984561

**Table 3:** The optimized calibration results of the internal parameters of the left camera based on the CLI-WPP algorithm.

Calibration Parameters	Calibration Results
$\mathrm{Focal}\;\mathrm{Length}\;[a_{x},a_{y}$]	[3297.40374, 3321.59384]
$\mathrm{Principal}\;\mathrm{Point}\;[u_{0},v_{0}$]	[579.399989, 430.634941]
$\mathrm{Distortion}\;\mathrm{Coefficient}\;[k_{1},k_{2}$]	[−0.01744174, 0.38125975]
Reprojection Error	0.06615037

**Table 4:** The optimized calibration results of the right camera’s internal parameters based on the CLI-WPP algorithm.

Calibration Parameters	Calibration Results
$\mathrm{Focal}\;\mathrm{Length}\;[a_{x},a_{y}$]	[3346.80389, 3344.06814]
$\mathrm{Principal}\;\mathrm{Point}\;[u_{0},v_{0}$]	[637.192839, 481.437970]
$\mathrm{Distortion}\;\mathrm{Coefficient}\;[k_{1},k_{2}$]	[−0.03579978, 0.50400146]
Reprojection Error	0.08240601

## Data Availability

The data presented in this study are available upon request from the corresponding author due to privacy concerns.
